# Supplementary material for: Loss of TRIM29 mitigates viral myocarditis by attenuating PERK-driven ER stress response in male mice
Source: Nat Commun. 2024 Apr 25;15:3481. doi: 10.1038/s41467-024-44745-x (PMC11045800; doi:10.1038/s41467-024-44745-x)
Supplement: Supplementary file 1 — Supplementary Information [file 41467_2024_44745_MOESM1_ESM.pdf]

Supplementary Information for:

# Loss of TRIM29 mitigates viral myocarditis by attenuating PERK-driven ER stress response in male mice

Junying Wang[1,4], Wenting Lu[1,4], Jerry Zhang[1,4], Yong Du[1,4], Mingli Fang[1], Ao Zhang[1], Gabriel Sungcad[1], Samantha Chon[1], Junji Xing[1,2,3,*]

[1]Department of Surgery and Immunobiology and Transplant Science Center, Houston Methodist Research Institute, Houston Methodist, Houston, TX 77030, USA

[2]Department of Cardiovascular Sciences, Houston Methodist Research Institute, Houston Methodist, Houston, TX 77030, USA

[3]Department of Surgery, Weill Cornell Medicine, Cornell University, New York, NY 10065, USA

[4]These authors contributed equally to this work.

*Correspondence: jxing@houstonmethodist.org **(J.X.)**

This document includes Supplementary Figures 1-9, and Supplementary Tables 1-3

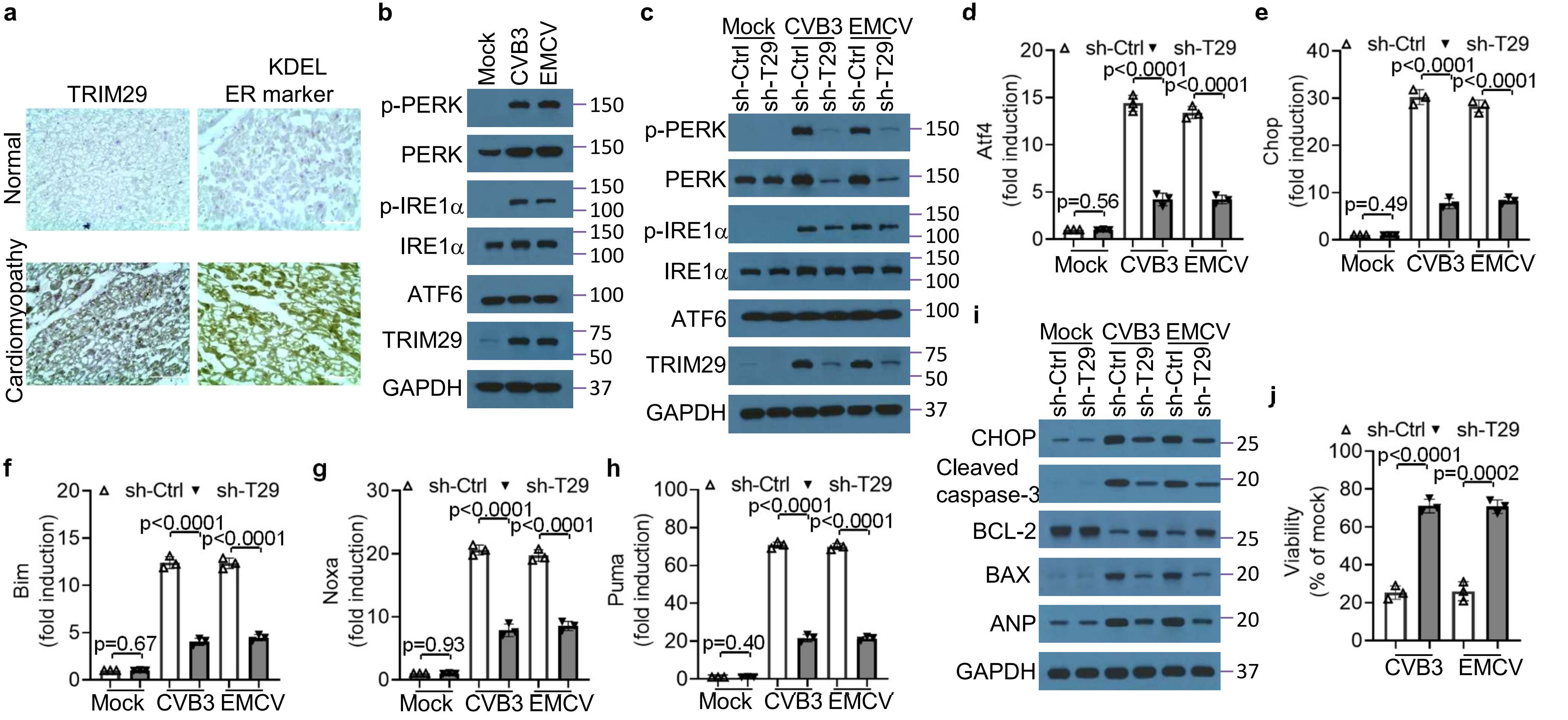


**Supplementary Fig. 1: TRIM29 knockdown inhibits PERK-mediated ER stress and apoptosis induced by cardiotropic viruses in human cardiomyocytes. a,** Immunohistochemistry (IHC) analysis of TRIM29 and ER marker KDEL expression in human heart tissues from healthy normal human adults and human patients with cardiomyopathy using an anti-KDEL ER marker antibody. Scale bars represent 100 μm. **b, c,** Immunoblot analysis of PERK, IRE1α, ATF6 and TRIM29 protein levels in AC16 human cardiomyocytes treated without (**b**) or with (**c**) an shRNA for TRIM29 knockdown expression (sh-T29) or a scrambled control shRNA (sh-Ctrl) followed by infection without (Mock) or with CVB3 and EMCV for 6 hours at a multiplicity of infection (MOI) of 1. **d-h,** RT-qPCR analysis of Atf4 (**d**), Chop (**e**), Bim (**f**), Noxa (**g**) and Puma (**h**) at the mRNA level in AC16 human cardiomyocytes treated with an shRNA for TRIM29 knockdown (sh-T29) or a scrambled control shRNA (sh-Ctrl) followed by infection without (Mock) or with CVB3 and EMCV for 3 hours at an MOI of 1. **i,** Immunoblot analysis of CHOP, cleaved caspase-3, BCL-2, BAX and ANP protein levels in AC16 human cardiomyocytes treated with an shRNA for TRIM29 knockdown (sh-T29) or a scrambled control shRNA (sh-Ctrl) followed by infection without (Mock) or with CVB3 and EMCV for 6 hours at an MOI of 1. **j,** Cell viability quantification analysis of AC16 human cardiomyocytes treated with an shRNA for TRIM29 knockdown (sh-T29) or a scrambled control shRNA (sh-Ctrl) followed by infection without or with CVB3 and EMCV for 12 hours at an MOI of 1 using the CellTiter-Glo assay. The luminescence intensity of the mock group was defined as 100%. Mock, shRNA-treated human cardiomyocytes without infection. Data are shown as the mean ±SD. Statistical significance was determined by a two-tailed, unpaired Student's t test. Data are representative of three independent experiments. Source data are provided as a Source Data file.

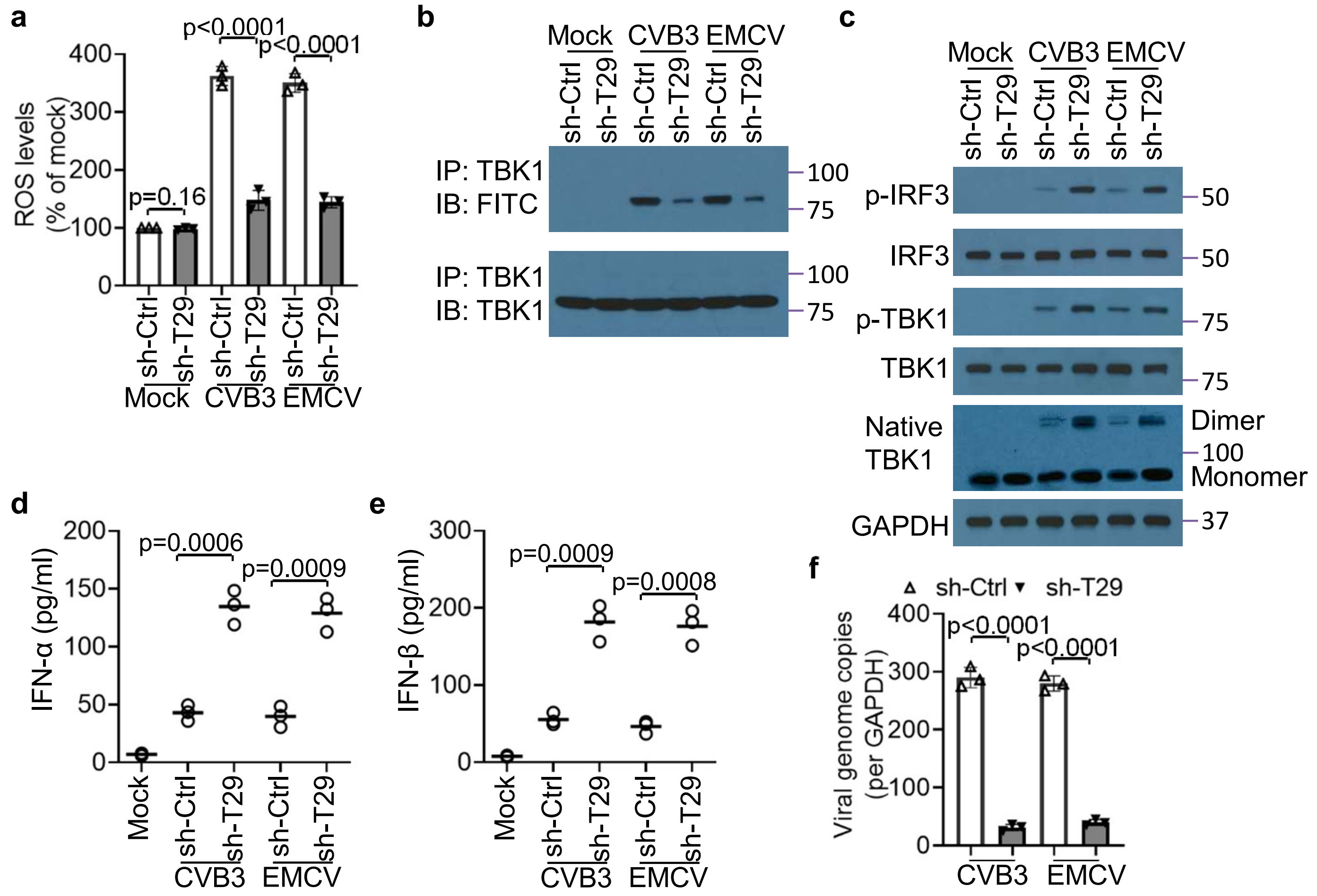


**Supplementary Fig. 2: TRIM29 knockdown unleashes type I interferon production to restrict cardiotropic viruses by relieving ROS-mediated TBK1 inhibition in human cardiomyocytes. a,** ROS production analysis of AC16 human cardiomyocytes treated with an shRNA for TRIM29 knockdown expression (sh-T29) or a scrambled control shRNA (sh-Ctrl) followed by infection without or with CVB3 and EMCV for 6 hours at an MOI of 1 using a DCFDA cellular ROS assay kit. The fluorescence intensity of the mock group was defined as 100%. **b,** Immunoblot analysis of TBK1 and FITC precipitated with anti-TBK1 from whole-cell lysates incubated with 5 μM 5-IAF for 1 hour labeling of free thiols in AC16 human cardiomyocytes treated with an shRNA for TRIM29 knockdown expression (sh-T29) or a scrambled control shRNA (sh-Ctrl) followed by infection without or with CVB3 and EMCV for 6 hours at an MOI of 5. **c,** Immunoblot analysis of IRF3, TBK1 and native TBK1 monomer and dimer in AC16 human cardiomyocytes treated with an shRNA for TRIM29 knockdown expression (sh-T29) or a scrambled control shRNA (sh-Ctrl) followed by infection without or with CVB3 and EMCV for 6 hours at an MOI of 5. **d, e,** ELISA of IFN-α (**d**) and IFN-β (**e**) production by AC16 human cardiomyocytes treated with an shRNA for TRIM29 knockdown expression (sh-T29) or a scrambled control shRNA (sh-Ctrl) followed by infection without or with CVB3 and EMCV for 16 hours at an MOI of 5. Each circle represents one value of the three biological replicates; small horizontal lines indicate the average of triplicates. **f,** Quantification of the expression of CVB3 and EMCV viral genome copies relative to GAPDH in AC16 human cardiomyocytes treated with an shRNA for TRIM29 knockdown expression (sh-T29) or a scrambled control shRNA (sh-Ctrl) followed by infection without or with CVB3 and EMCV for 6 hours at an MOI of 1. Mock, shRNA-treated human cardiomyocytes without infection. Data are shown as the mean ±SD. Statistical significance was determined by a two-tailed, unpaired Student's t test. Data are representative of three independent experiments. Source data are provided as a Source Data file.

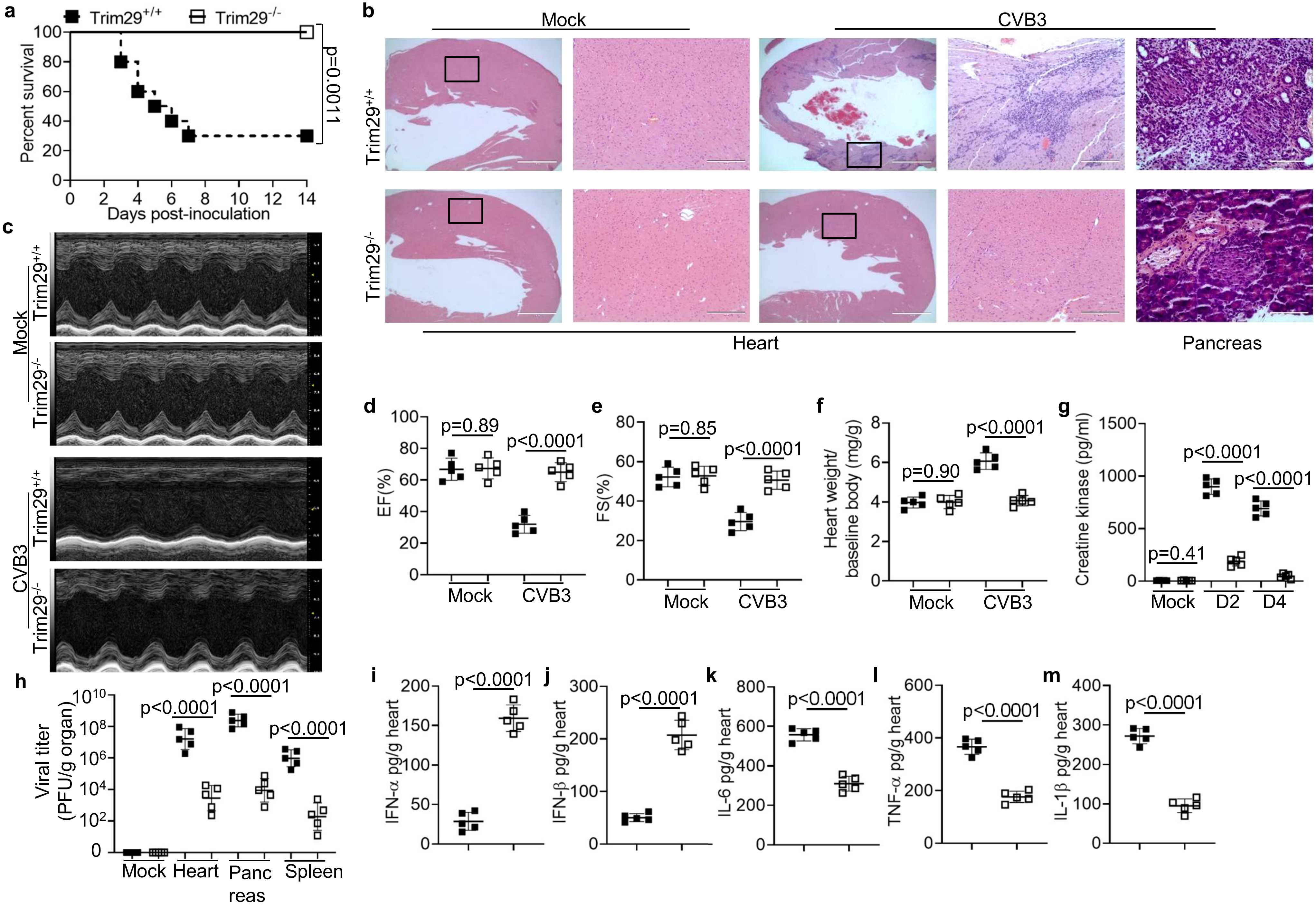

a
Trim29+/+
Trim29-/-
Percent survival
Days post-inoculation
p=0.0011
b
Mock
CVB3
Trim29+/+
Trim29-/-
Heart
Pancreas
c
Mock
CVB3
Trim29+/+
Trim29-/-
d
EF(%)
p=0.89
p<0.0001
Mock
CVB3
e
FS(%)
p=0.85
p<0.0001
f
Heart weight/ baseline body (mg/g)
p=0.90
p<0.0001
g
Creatine kinase (pg/ml)
p=0.41
p<0.0001
p<0.0001
D2
D4
h
Viral titer (PFU/g organ)
p<0.0001
p<0.0001
p<0.0001
Mock
Heart
Panc reas
Spleen
i
IFN-α pg/g heart
p<0.0001
j
IFN-β pg/g heart
p<0.0001
k
IL-6 pg/g heart
p<0.0001
l
TNF-α pg/g heart
p<0.0001
m
IL-1β pg/g heart
p<0.0001

**Supplementary Fig. 3: Knockout of TRIM29 protects mice from CVB3 induced myocarditis *in vivo*. a,** Survival of *Trim29*$^{+/+}$ and *Trim29*$^{-/-}$ mice after intraperitoneal infection with CVB3 ($1\times10^7$ PFU per mouse) (n=10 per group). **b,** Hematoxylin and eosin (H&E)-staining of heart sections from *Trim29*$^{+/+}$ and *Trim29*$^{-/-}$ mice after intraperitoneal infection without (Mock) or with CVB3 ($1\times10^7$ PFU per mouse) for 4 days. Scale bars represent 1000 μm for original images and 400 μm for enlarged images. **c,** Representative M-mode echocardiography images of hearts from *Trim29*$^{+/+}$ and *Trim29*$^{-/-}$ mice on day 4 after CVB3 infection. **d,e,** Cardiac function analysis of ejection fraction (EF) (**d**) and fractional shortening (FS) (**e**) of hearts from mice as in (**c**) (n=5 per group). **f,** Assessment of heart weight/baseline body weight in *Trim29*$^{+/+}$ and *Trim29*$^{-/-}$ mice (n = 5 per group) on day 0 or day 6 after CVB3 infection. **g,h,** ELISA of creatine kinase production in sera (**g**) and viral titers in homogenates of hearts (**h**) from *Trim29*$^{+/+}$ and *Trim29*$^{-/-}$ mice on day 0 (Mock), day 2 and day 4 after CVB3 infection (n=5 per group). **i-m,** ELISA of IFN-α (**i**), IFN-β (**j**) IL-6 (**k**), TNF-α (**l**) and IL-1β (**m**) in hearts from *Trim29*$^{+/+}$ and *Trim29*$^{-/-}$ mice on day 2 after CVB3 infection (n = 5 per group). Data are shown as the mean ± SD. Statistical significance was determined by a two-tailed, unpaired Student's t test and Gehan-Breslow-Wilcoxon test for survival analysis. Data are representative of three independent experiments. Source data are provided as a Source Data file.

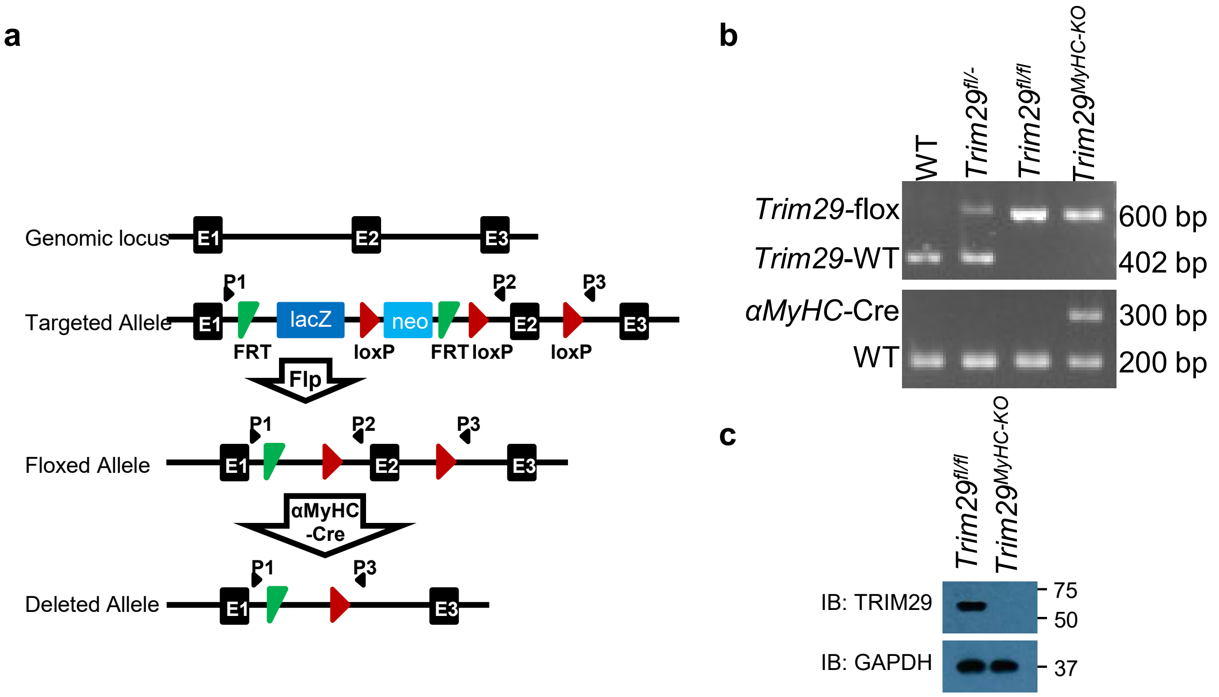


**Supplementary Fig. 4: Generation of cardiomyocyte specific Trim29 knockout mice. a**, Schematic picture of Trim29 gene targeting using an FRT-LoxP vector, showing the exons 1 to 3 of Trim29 gene. Targeted mice were crossed with FRT deleter (Rosa26-FLPe) mice to generate *Trim29*-floxed (*Trim29*$^{fl/fl}$) mice, which were further crossed with αMyHC-Cre transgenic mice to generate cardiomyocyte-specific Trim29-knockout mice, *Trim29*$^{fl/fl}$; *αMyHC-Cre* (*Trim29*$^{MyHC\text{-}KO}$). **b**, Genotyping PCR to amplify the *Trim29-flox* (using P1/P2 primer pair) and WT (using P1/P2 primer pair) alleles (top), or the *αMyHC*-Cre (using 16775/oIMR9074 primer pair) and WT (using 16775/16776 primer pair) alleles (bottom). **c,** Immunoblot (IB) of TRIM29 in mouse primary neonatal cardiomyocytes from wild-type *Trim29*$^{fl/fl}$ and *Trim29*$^{MyHC\text{-}KO}$ mice with CVB3 infection for 6 hours at an MOI of 1. The position of protein markers (shown in kDa) is indicated on the right. Data are representative of three independent experiments. Source data are provided as a Source Data file.

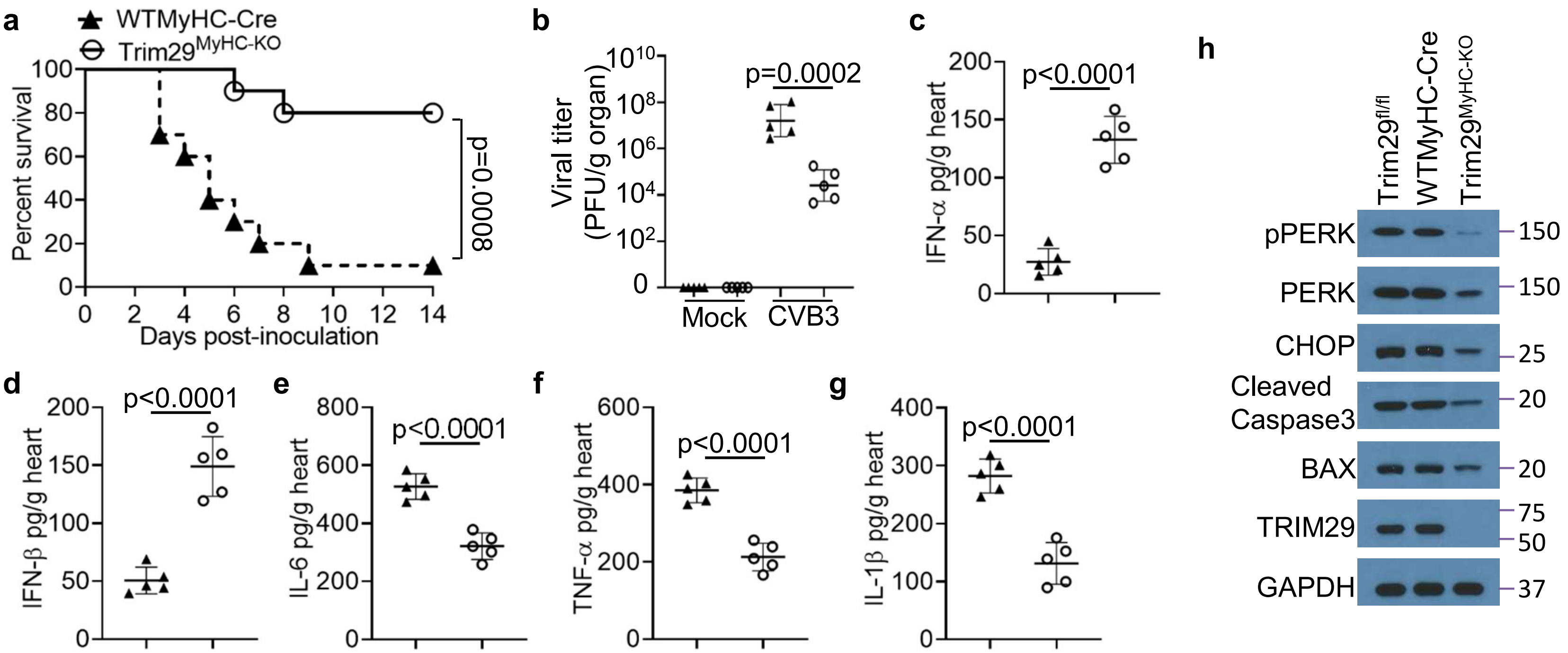


**Supplementary Fig. 5: Cardiomyocyte-specific TRIM29 deficiency protects mice from CVB3 induced myocarditis and PERK-mediated ER stress and apoptosis compared with WTMyHC-Cre mice *in vivo*. a,** Survival of WTMyHC-Cre and *Trim29*$^{MyHC\text{-}KO}$ mice after intraperitoneal infection with CVB3 ($1\times10^7$ PFU per mouse) (n=10 per group). **b,** Viral titers in homogenates of hearts from WTMyHC-Cre and *Trim29*$^{MyHC\text{-}KO}$ mice on day 0 (Mock) and day 4 after CVB3 infection (n=5 per group). **c-g,** ELISA of IFN-α (**c**), IFN-β (**d**) IL-6 (**e**), TNF-α (**f**) and IL-1β (**g**) in hearts from WTMyHC-Cre and *Trim29*$^{MyHC\text{-}KO}$ mice on day 2 after CVB3 infection (n = 5 per group). **h,** Immunoblot analysis of PERK, CHOP, cleaved caspase-3, BAX and TRIM29 protein levels in cardiomyocytes isolated from wild-type Trim29$^{fl/fl}$, WTMyHC-Cre or Trim29$^{MyHC\text{-}KO}$ mice infected with CVB3 for 2 days. Data are shown as the mean ± SD. Statistical significance was determined by a two-tailed, unpaired Student's t test and Gehan-Breslow-Wilcoxon test for survival analysis. Data are representative of three independent experiments. Source data are provided as a Source Data file.

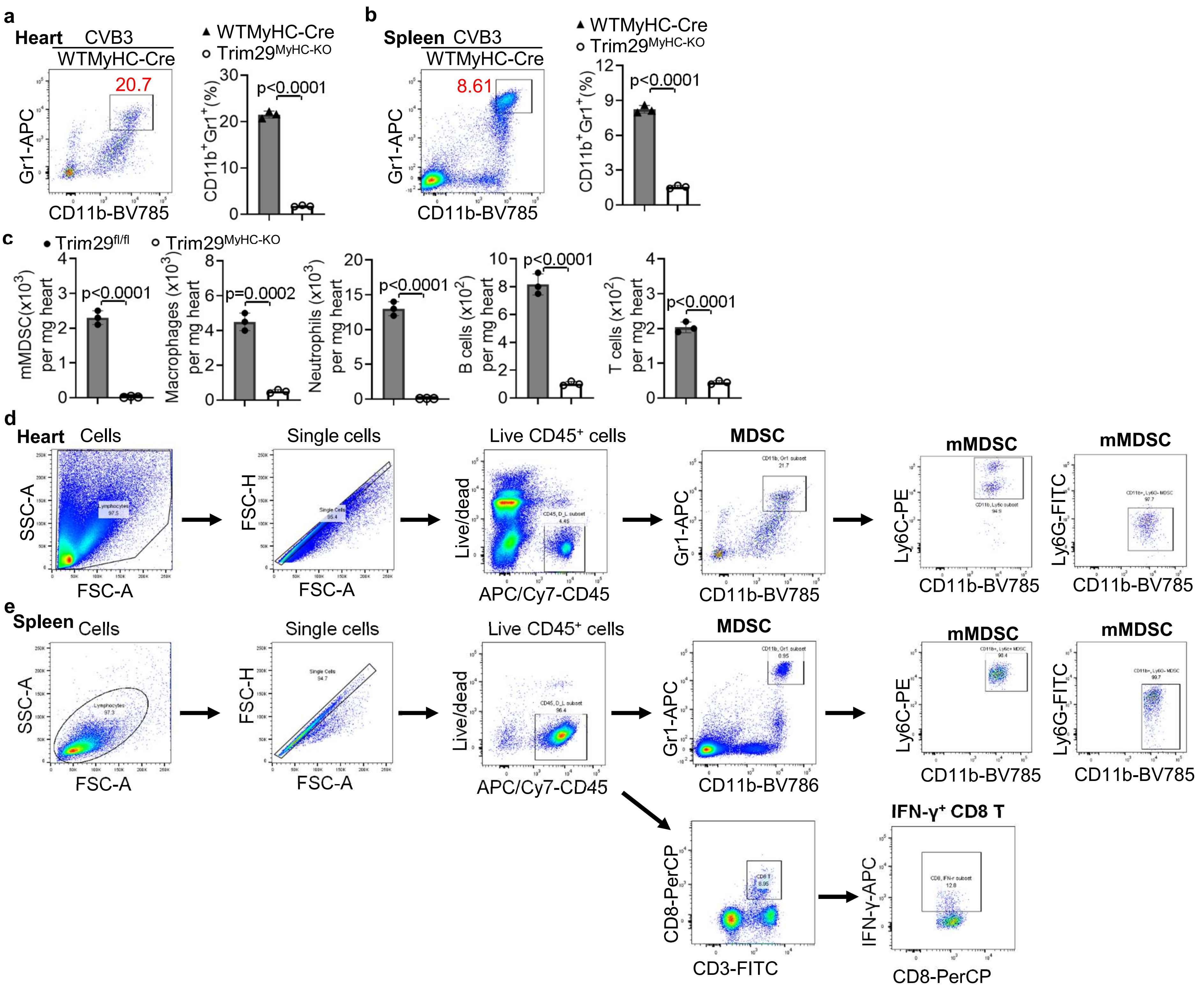

a
Heart CVB3
WTMyHC-Cre
20.7
Gr1-APC
CD11b-BV785
▲ WTMyHC-Cre
○ Trim29MyHC-KO
CD11b+Gr1+(%)
p<0.0001
b
Spleen CVB3
WTMyHC-Cre
8.61
Gr1-APC
CD11b-BV785
▲ WTMyHC-Cre
○ Trim29MyHC-KO
CD11b+Gr1+(%)
p<0.0001
c
● Trim29fl/fl
○ Trim29MyHC-KO
mMDSC(x10^3) per mg heart
p<0.0001
Macrophages (x10^3) per mg heart
p=0.0002
Neutrophils (x10^3) per mg heart
p<0.0001
B cells (x10^2) per mg heart
p<0.0001
T cells (x10^2) per mg heart
p<0.0001
d
Heart Cells
SSC-A
FSC-A
Single cells
FSC-H
FSC-A
Live CD45+ cells
Live/dead
APC/Cy7-CD45
MDSC
Gr1-APC
CD11b-BV785
mMDSC
Ly6C-PE
CD11b-BV785
mMDSC
Ly6G-FITC
CD11b-BV785
e
Spleen Cells
SSC-A
FSC-A
Single cells
FSC-H
FSC-A
Live CD45+ cells
Live/dead
APC/Cy7-CD45
MDSC
Gr1-APC
CD11b-BV786
mMDSC
Ly6C-PE
CD11b-BV785
mMDSC
Ly6G-FITC
CD11b-BV785
CD8-PerCP
CD3-FITC
IFN-γ+ CD8 T
IFN-γ-APC
CD8-PerCP

**Supplementary Fig. 6: Cardiomyocyte-specific TRIM29 deficiency reduces PERK-mediated mMDSC and other immune cells during viral myocarditis compared with WTMyHC-Cre mice *in vivo*. a,b**, Flow cytometry and quantification analysis of mouse mMDSC cells of heart (**a**) infiltrated immune cells and spleen (**b**) immune cells from WTMyHC-Cre and *Trim29*$^{\text{MyHC-KO}}$ mice infected with CVB3 for 2 days using CD11b-BV785 and Gr1-APC antibodies. **c**, Flow cytometry cell number quantification analysis of mouse mMDSC, macrophages, neutrophils, B cells and T cells of heart infiltrated immune cells from *Trim29*$^{\text{fl/fl}}$ and *Trim29*$^{\text{MyHC-KO}}$ mice infected with CVB3 for 2 days. **d,e,** Representative FACS plots showing the gating strategy for analyzing mMDSC and IFN-$\gamma^{+}$ CD8 T cell populations in heart (**d**) or spleen (**e**) of the mice. Flow cytometry data were acquired on an LSR-II flow cytometer (Beckton Dickinson) and analyzed using FlowJo v10 software (Tree Star). Data are shown as the mean ± SD. Statistical significance was determined by a two-tailed, unpaired Student's t test. Data are representative of three independent experiments. Source data are provided as a Source Data file.

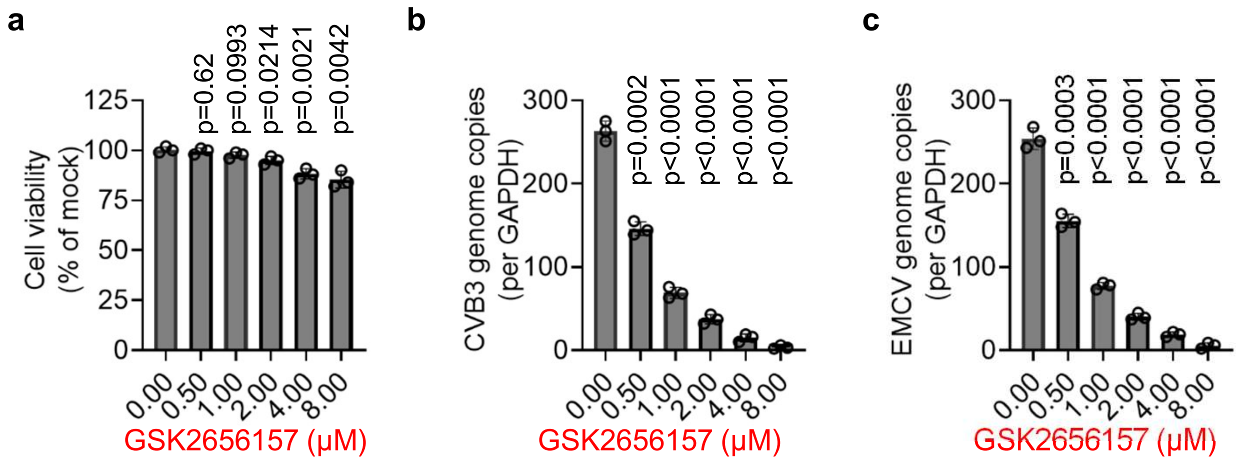


**Supplementary Fig. 7: PERK inhibitor reduces replication of cardiotropic viruses in mouse cardiomyocytes. a,** Cell viability quantification analysis of mouse primary neonatal cardiomyocytes from *Trim29*$^{+/+}$ mice treated with the indicated concentrations of PERK inhibitor GSK2656157 for 24 hours using CellTiter-Glo assay. **b,c,** Quantification of expression of CVB3 (**b**) and EMCV (**c**) viral genome copies relative to GAPDH in mouse neonatal cardiomyocytes from *Trim29*$^{+/+}$ mice infected with CVB3 (**b**) and EMCV (**c**) at an MOI of 0.1 and treated with the indicated concentrations of PERK inhibitor GSK2656157 for 24 hours. Data are shown as the mean ± SD. Statistical difference between groups was determined by a two-tailed, unpaired Student's t test. Data are representative of three independent experiments. Source data are provided as a Source Data file.

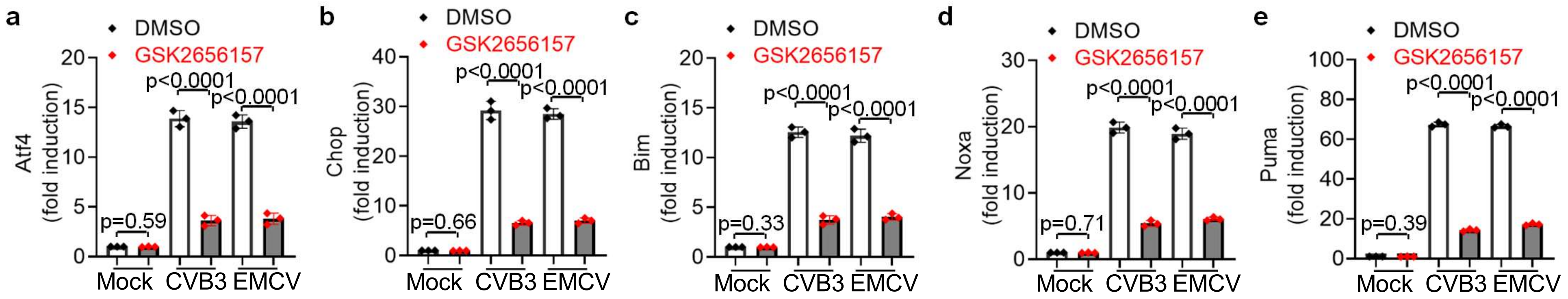


**Supplementary Fig. 8: PERK inhibitor reduces apoptosis induced by cardiotropic viruses in cardiomyocytes. a-e**, The RT-qPCR analysis of Atf4 **(a)**, Chop **(b)**, Bim **(c)**, Noxa **(d)** and Puma **(e)** at the mRNA level in mouse primary neonatal cardiomyocytes from *Trim29*$^{+/+}$ mice by infection without or with CVB3 and EMCV at an MOI of 1 and treated with PERK inhibitor GSK2656157 or DMSO for 6 hours. Data are shown as the mean ± SD. Statistical significance was determined by a two-tailed, unpaired Student's t test. Mock, mouse cardiomyocytes without infection. Data are representative of three independent experiments. Source data are provided as a Source Data file.

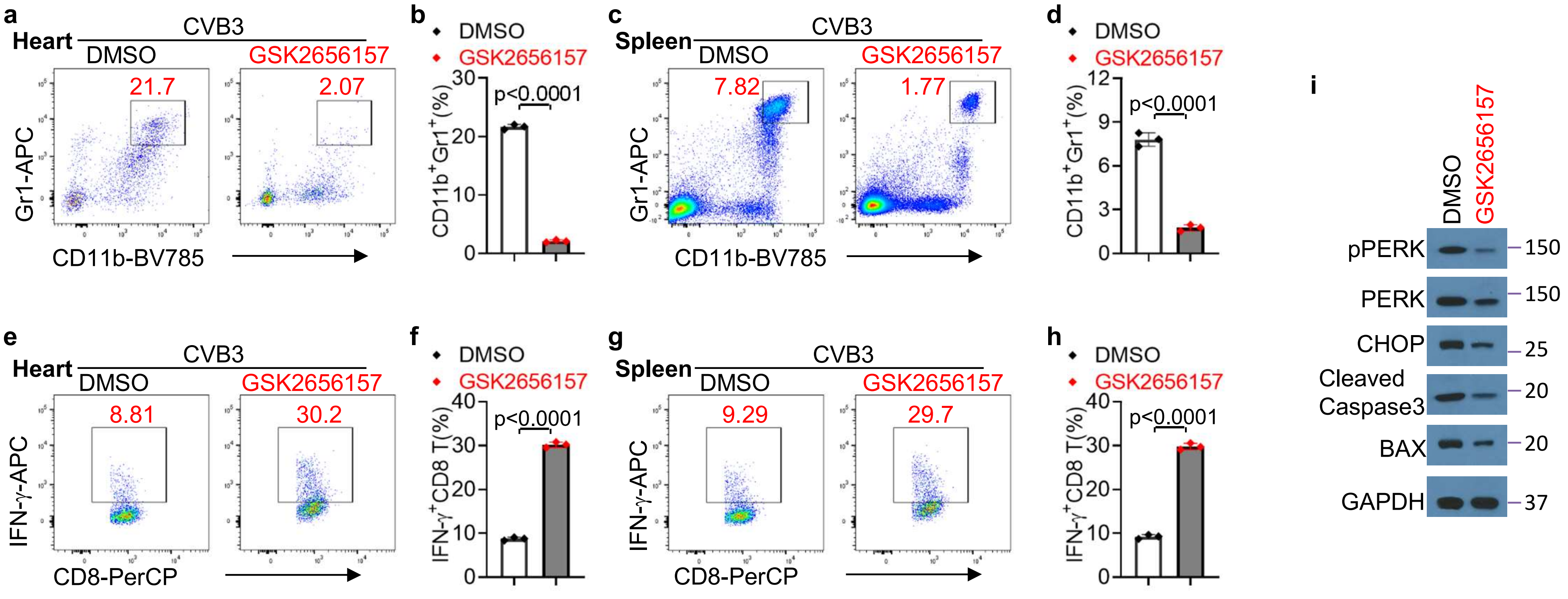


**Supplementary Fig. 9: PERK inhibitor reduces PERK mediated mMDSC to enhance antiviral CD8 T cell functions and PERK mediated ER stress and apoptosis during viral myocarditis *in vivo*. a-d,** Flow cytometry (**a**,**c**) and quantification (**b**,**d**) analysis of mouse mMDSC ($CD11b^+Gr1^+$) cells of heart infiltrated immune cells (**a**,**b**) and spleen immune cells (**c**,**d**) from wild-type mice infected with CVB3 and treated with PERK inhibitor GSK2656157 or DMSO for 2 days using CD11b-BV785 and Gr1-APC antibodies. **e-h,** Flow cytometry (**e**,**g**) and quantification (**f**,**h**) analysis of mouse IFN-γ producing CD8 T cells (IFN-$\gamma^+$ CD8 T cells) of heart infiltrated lymphocytes (**e**,**f**) and spleen lymphocytes (**g**,**h**) from wild-type mice infected with CVB3 and treated with PERK inhibitor GSK2656157 or DMSO for 2 days using CD8-PerCP and IFN-γ-APC antibodies. **i,** Immunoblot analysis of PERK, CHOP, cleaved caspase-3, and BAX protein levels in cardiomyocytes isolated from wild-type mice infected with CVB3 and treated with PERK inhibitor GSK2656157 or DMSO for 2 days. Flow cytometry data were acquired on an LSR-II flow cytometer (Beckton Dickinson) and analyzed using FlowJo v10 software (Tree Star). Data are shown as the mean ± SD. Statistical significance was determined by a two-tailed, unpaired Student's t test. Data are representative of three independent experiments. Source data are provided as a Source Data file.

## Supplementary Table 1: PERK is in the TRIM29-binding protein complex.

| NCBI gi no. | Protein Name | Peptide Hits |
|---|---|---|
| 160333881 | tripartite motif-containing protein 29 [Mus musculus] | 487 |
| 255760028 | hematopoietic lineage cell-specific protein [Mus musculus] | 317 |
| 6671509 | actin, cytoplasmic 1 [Mus musculus] | 254 |
| 255958292 | delta-1-pyrroline-5-carboxylate synthase isoform 1 [Mus musculus] | 228 |
| 7106439 | tubulin beta-5 chain [Mus musculus] | 214 |
| 31981939 | tubulin beta-4A chain [Mus musculus] | 185 |
| 114326446 | myosin-9 isoform 1 [Mus musculus] | 170 |
| 14192922 | actin, alpha cardiac muscle 1 [Mus musculus] | 160 |
| 31981690 | heat shock cognate 71 kDa protein [Mus musculus] | 143 |
| 6755372 | 40S ribosomal protein S3 [Mus musculus] | 114 |
| 255982600 | centrosome-associated protein 350 [Mus musculus] | 108 |
| 19705578 | V-type proton ATPase subunit B, brain isoform [Mus musculus] | 94 |
| 158186704 | heterogeneous nuclear ribonucleoprotein M isoform b [Mus musculus] | 92 |
| 183396771 | 60 kDa heat shock protein, mitochondrial [Mus musculus] | 82 |
| 255003735 | 60S ribosomal protein L10a [Mus musculus] | 76 |
| 22094075 | ADP/ATP translocase 2 [Mus musculus] | 70 |
| 1720416963 | eukaryotic translation initiation factor 2-alpha kinase 3 isoform 1 [Mus musculus] (PERK) | 66 |
| 254540166 | 78 kDa glucose-regulated protein precursor [Mus musculus] | 60 |
| 21312564 | calponin-3 [Mus musculus] | 55 |
| 160333923 | heterogeneous nuclear ribonucleoprotein U [Mus musculus] | 48 |
| 110835723 | putative pre-mRNA-splicing factor ATP-dependent RNA helicase DHX15 isoform 2 [Mus musculus] | 44 |
| 169234614 | immune-responsive gene 1 protein [Mus musculus] | 40 |
| 6753620 | ATP-dependent RNA helicase DDX3X [Mus musculus] | 34 |
| 294862303 | Ubiquitin-conjugating enzyme E2I, isoform 1 [Mus musculus] (Ubc9) | 26 |
| 113205073 | E3 ubiquitin-protein ligase AMFR [Mus musculus] | 25 |
| 153792534 | E3 SUMO-protein ligase RanBP2 [Mus musculus] | 21 |
| 115511018 | probable ubiquitin carboxyl-terminal hydrolase FAF-X [Mus musculus] | 19 |
| 168480106 | zinc finger RNA-binding protein [Mus musculus] | 18 |
| 110625761 | AFG3-like protein 2 [Mus musculus] | 17 |
| 33859662 | synaptic vesicle membrane protein VAT-1 homolog [Mus musculus] | 16 |
| 19882201 | 26S proteasome non-ATPase regulatory subunit 2 [Mus musculus] | 15 |
| 130507685 | unconventionnal myosin-X [Mus musculus] | 14 |
| 29126205 | 3-ketoacyl-CoA thiolase, mitochondrial [Mus musculus] | 13 |
| 13386026 | UPF0568 protein C14orf166 homolog [Mus musculus] | 12 |
| 228008337 | 26S protease regulatory subunit 6A [Mus musculus] | 11 |
| 118403314 | RNA-binding protein 39 [Mus musculus] | 10 |
| 31543976 | 14-3-3 protein gamma [Mus musculus] | 9 |
| 124053459 | unconventional myosin-IXb isoform 3 [Mus musculus] | 8 |
| 6755210 | 26S proteasome non-ATPase regulatory subunit 13 [Mus musculus] | 7 |
| 34368584 | zinc finger CCCH domain-containing protein 15 [Mus musculus] | 6 |
| 6679501 | 26S protease regulatory subunit 4 [Mus musculus] | 5 |
| 71534295 | endoplasmic reticulum mannosyl-oligosaccharide 1,2-alpha-mannosidase [Mus musculus] | 4 |
| 261823966 | zinc finger protein 638 isoform 1 [Mus musculus] | 4 |

| | | |
|---|---|---|
| 281332108 | 2'-5'-oligoadenylate synthase 1A [Mus musculus] | 3 |
| 21312167 | hydroxyacylglutathione hydrolase-like protein [Mus musculus] | 2 |
| 11037792 | serine/threonine-protein kinase Nek7 [Mus musculus] | 2 |
| 83649741 | WD repeat-containing protein 18 [Mus musculus] | 2 |

Mouse neonatal cardiomyocytes lysate was prepared, followed by anti-TRIM29 immunoprecipitation and protein sequencing by liquid chromatography-mass spectrometry. NCBI gi no: unique protein identification number; Hits: the number of peptides ions matched that associated protein.

**Supplementary Table 2: The potential SUMOylation sites at the PERK molecule and the putative SUMO-interacting motif (SIM) in the TRIM29 molecule.**

Mouse PERK protein sequence (1114 AA):

MERATRPGPRALLLLLFLLLGCAAGISAVAPARSLLAPASETVFGLGAAAAPTSAARVPAVATAEVTVEDAEALPAA
AGEPESRATEPDDDVELRPRGRSLVIISTLDGRIAALDAENDGKKQWDLDVGSGSLVSSSLSKPEVFGNKMIIPSLD
GDLFQWDRDRESMEAVPFTVESLLESSYKFGDDVVLVGGKSLTTYGLSAYSGKLRYICSALGCRRWDSDEMEEEEDI
LLLQRTQKTVRAVGPRSGSEKWNFSVGHFELRYIPDMETRAGFIESTFKPGGNKEDSKIISDVEEQEATMLDTVIKV
SVADWKVMAFSRKGGRLEWEYQFCTPIASAWLVRDGKVIPISLFDDTSYTASEEALGDEEDIVEAARGATENSVYLG
MYRGQLYLQSSVRVSEKFPTSPKALESVNGENAIIPLPTIKWKPLIHSPSRTPVLVGSDEFDKCLSNDKYSHEEYSN
GALSILQYPYDNGYYLPYYKRERNKRSTQITVRFLDSPHYSKNIRKKDPILLLHWWKEIFGTILLCIVATTFIVRRL
FHPQPHRQRKESETQCQTESKYDSVSADVSDNSWNDMKYSGYVSRYLTDFEPIQCMGRGGFGVVFEAKNKVDDCNYA
IKRIRLPNRELAREKVMREVKALAKLEHPGIVRYFNAWLETPPEKWQEEMDEIWLKDESTDWPLSSPSPMDAPSVKI
RRMDPFSTKEQIEVIAPSPERSRSFSVGISCGQTSSSESQFSPLEFSGTDCGDNSDSADAAYNLQDSCLTDCEDVED
GTVDGNDEGHSFELCPSEASPYTRSREGTSSSIVFEDSGCGNASSKEEPRGNRLHDGNHYVNKLTDLKCSSSRSSSE
ATTLSTSPTRPTTLSLDFTKNTVGQLQPSSPKVYLYIQMQLCRKENLKDWMNRRCSLEDREHGVCLHIFLQIAEAVE
FLHSKGLMHRDLKPSNIFFTMDDVVKVGDFGLVTAMDQDEEEQTVLTPMPAYATHTGQVGTKLYMSPEQIHGNNYSH
KVDIFSLGLILFELLYPFSTQMERVRILTDVRNLKFPLLFTQKYPQEHMMVQDMLSPSPTERPEATDIIENAIFENL
EFPGKTVLRQRSRSMSSSGTKHSRQPSCSYSPLPGN

| Results for putatifs SUMO site | | | | | | | | |
|---|---|---|---|---|---|---|---|---|
| Position K | Sequence | Best PS | Consensus direct | | | Consensus Inverted | | |
| | | | Type | PSd | DB Hit | Type | PSi | DB Hit |
| K140 | SGSLVSSSLSKPEVFGNKMII | None | None | None | 1 | None | None | 1 |
| K194 | FGDDVVLVGGKSLTTYGLSAY | None | None | None | 1 | None | None | |
| K289 | FKPGGNKEDSKIISDVEEQEA | Low | None | None | | Strong consensus inv | Low | |
| K307 | QEATMLDTVIKVSVADWKVMA | None | None | None | | None | None | 1 |
| K314 | TVIKVSVADWKVMAFSRKGGR | High | None | None | | Strong consensus inv | High | |
| K321 | ADWKVMAFSRKGGRLEWEYQF | None | None | None | 1 | None | None | 1 |
| K345 | IASAWLVRDGKVIPISLFDDT | High | None | None | | Strong consensus inv | High | |
| K482 | YDNGYYLPYYKRERNKRSTQI | None | Weak Consensus | None | 1 | None | None | |
| K560 | ESETQCQTESKYDSVSADVSD | None | None | None | | Weak consensus inv | None | |
| K577 | DVSDNSWNDMKYSGYVSRYLT | None | None | None | | Weak consensus inv | None | |
| K618 | NKVDDCNYAIKRIRLPNRELA | None | None | None | 1 | None | None | |
| K637 | LAREKVMREVKALAKLEHPGI | Low | None | None | 1 | Consensus inv | Low | 1 |
| K641 | KVMREVKALAKLEHPGIVRYF | Low | HCSM | Low | 2 | None | None | 1 |
| K672 | WQEEMDEIWLKDESTDWPLSS | Low | Strong Consensus | Low | 6 | None | None | |
| K929 | IAEAVEFLHSKGLMHRDLKPS | None | None | None | 1 | None | None | |
| K937 | HSKGLMHRDLKPSNIFFTMDD | Low | None | None | | Consensus inv | Low | 2 |
| K1036 | VRILTDVRNLKFPLLFTQKYP | None | None | None | 1 | None | None | |

The possibility of SUMOylation sites in PERK (bottom panel) was predicted from the amino acid sequence of PERK (top panel) by “JASSA: Joined Advanced Sumoylation Site and Sim Analyser” (http://www.jassa.fr/).

Mouse TRIM29 protein sequence (587 AA):

MEGADACRSNGASPEARDTRSPPGPSGSLENGTKADSKDTKTTNGHSGEVTEGKTLGSALKSGEGKSGLF
SSNEWRRPIIQFVESVDDKGSSYFSMDSAEGRRSPYAGLQLGASKKPPVTFAEKGELRKSIFSEPRKPTV
TIVEPGEVRRNSYPRADSSLLARAKSGSEEVLCDSCIGNKQKAVKSCLVCQASFCELHLKPHLEGAAFRD
HQLLEPIRDFEARKCPLHGKTMELFCQTDQTCICYLCMFQEHKNHSTVTVEEAKAEKETELSLQKEQLQL
KIIEIEDDVEKWQKEKDRIKSFTTNEKAILEQNFRDLVRELEKQKEEVRAALEQREQDAVDQVKVIVDAL
DERAKVLHEDKQTREQLHNISDSVLFLQEFGALMSNYSLPPPLPTYHVLLEGEGLGQSLGNCKDDLLNVC
MRHVEKMCKADLSRNFIERNHMENGGDHRYMNSYTSSYGNEWSTPDTMKRYSMYLTPKGGGRTSYQPSSP
SRLSKETNQKNFNNLYGTKGNYTSRVWEYTSTVQNSEDMPTVQGNSSFSLKGFPSLLRSQVPKAQPQTWK
SGKQTLLSHYRPFYVNKGSGIGSNEAP

**Results for putatifs SIM** [PSmax=38.183 | Cut-off=0.269]

| Position site | Sequence | Type | α/S stretch | PS | DB Hit |
|---|---|---|---|---|---|
| AA 140-143 | FSEPRKPTVTIVEPGEVRRN | SIM Type α | [N][SIM][N] | 0.808 | |
| AA 282-288 | QKEQLQLKIIEIEDDVEKWQ | SIM Type a | [N][SIM][Y] | 2.632 | 1 |

The putative SUMO-interacting motif (SIM) in TRIM29 (bottom panel) was predicted from the amino acid sequence of TRIM29 (top panel) by “JASSA: Joined Advanced Sumoylation Site and Sim Analyser” (http://www.jassa.fr/).

**Supplementary Table 3: Primers for qRT-PCR and PCR used in this study.**

| Gene | Sequence |
|---|---|
| qRT-PCR | |
| Human Gapdh | F: 5'- ATTCCACCCATGGCAAATTC -3' |
| | R: 5'- CGCTCCTGGAAGATGGTGAT -3' |
| Human Atf4 | F: 5'- GTTCTCCAGCGACAAGGCTA -3' |
| | R: 5'- ATCCTGCTTGCTGTTGTTGG -3' |
| Human Chop | F: 5'- AGAACCAGGAAACGGAAACAGA -3' |
| | R: 5'- TCTCCTTCATGCGCTGCTTT -3' |
| Human Bim | F: 5'- GGCCCCTACCTCCCTACA -3' |
| | R: 5'- GGGGTTTGTGTTGATTTGTCA -3' |
| Human Noxa | F: 5'- GTGCCCTTGGAAACGGAAGA -3' |
| | R: 5'- CCAGCCGCCCAGTCTAATCA -3' |
| Human Puma | F: 5'- CAGACTGTGAATCCTGTGCT -3' |
| | R: 5'- ACAGTATCTTACAGGCTGGG -3' |
| Mouse Gapdh | F: 5'- AGGTCGGTGTGAACGGATTTG -3' |
| | R: 5'- TGTAGACCATGTAGTTGAGGTCA -3' |
| Mouse Atf4 | F: 5'- TCGATGCTCTGTTTCGAATG -3' |
| | R: 5'- AGAATGTAAAGGGGGCAACC -3' |
| Mouse Chop | F: 5'- GTCCCTAGCTTGGCTGACAGA -3' |
| | R: 5'- TGGAGAGCGAGGGCTTTG -3' |
| Mouse Bim | F: 5'- GAGATACGGATTGCACAGGA -3' |
| | R: 5'- TCAGCCTCGCGGTAATCATT -3' |
| Mouse Noxa | F: 5'- GAGTGCACCGGACATAACTG -3' |
| | R: 5'- CTCGTCCTTCAAGTCTGCTG -3' |
| Mouse Puma | F: 5'- ATGGCGGACGACCTCAAC -3' |
| | R: 5'- AGTCCCATGAAGAGATTGTACATGAC -3' |
| Mouse genotype PCR | |
| Trim29 P1 | 5'- TCCCATCTTGCCTACACTGC -3' |
| Trim29 P2 | 5'- TCTGCTGGGCTCTGTGCTAC -3' |
| Trim29 P3 | 5'- TCGTGGTATCGTTATGCGCC -3' |
| αMhHC-Cre F | 5'-ATGACAGACAGATCCCTCCTATCTCC -3' |
| αMhHC-Cre R | 5'-CTCATCACTCGTTGCATCATCGAC -3' |
| Positive control F | 5'-CAAATGTTGCTTGTCTGGTG -3' |
| Positive control F | 5'-GTCAGTCGAGTGCACAGTTT -3' |
