# Supplementary material for: Loss of TRIM29 mitigates viral myocarditis by attenuating PERK-driven ER stress response in male mice
Source: Nat Commun. 2024 Apr 25;15:3481. doi: 10.1038/s41467-024-44745-x (PMC11045800; doi:10.1038/s41467-024-44745-x)
Supplement: Supplementary file 3 — Reporting Summary [file 41467_2024_44745_MOESM3_ESM.pdf]

nature portfolio

Corresponding author(s): Junji Xing

Last updated by author(s): Dec 8, 2023

# Reporting Summary

Nature Portfolio wishes to improve the reproducibility of the work that we publish. This form provides structure for consistency and transparency in reporting. For further information on Nature Portfolio policies, see our Editorial Policies and the Editorial Policy Checklist.

## Statistics

For all statistical analyses, confirm that the following items are present in the figure legend, table legend, main text, or Methods section.

| n/a | Confirmed | |
|---|---|---|
| ☐ | ☒ | The exact sample size (*n*) for each experimental group/condition, given as a discrete number and unit of measurement |
| ☐ | ☒ | A statement on whether measurements were taken from distinct samples or whether the same sample was measured repeatedly |
| ☐ | ☒ | The statistical test(s) used AND whether they are one- or two-sided<br>*Only common tests should be described solely by name; describe more complex techniques in the Methods section.* |
| ☒ | ☐ | A description of all covariates tested |
| ☒ | ☐ | A description of any assumptions or corrections, such as tests of normality and adjustment for multiple comparisons |
| ☐ | ☒ | A full description of the statistical parameters including central tendency (e.g. means) or other basic estimates (e.g. regression coefficient) AND variation (e.g. standard deviation) or associated estimates of uncertainty (e.g. confidence intervals) |
| ☐ | ☒ | For null hypothesis testing, the test statistic (e.g. *F*, *t*, *r*) with confidence intervals, effect sizes, degrees of freedom and *P* value noted<br>*Give P values as exact values whenever suitable.* |
| ☒ | ☐ | For Bayesian analysis, information on the choice of priors and Markov chain Monte Carlo settings |
| ☒ | ☐ | For hierarchical and complex designs, identification of the appropriate level for tests and full reporting of outcomes |
| ☒ | ☐ | Estimates of effect sizes (e.g. Cohen's *d*, Pearson's *r*), indicating how they were calculated |

*Our web collection on statistics for biologists contains articles on many of the points above.*

## Software and code

Policy information about availability of computer code

| | |
|---|---|
| Data collection | Cell viability, ROS measurement and ELISA: Synergy H1 Hybrid Multi-Mode Microplate Reader (BioTek INC),<br>Quantitative RT-PCR: CFX96 Touch Real-Time PCR Detection System (Bio-Rad),<br>Echocardiography measurement: Vevo 2100 system (VisualSonics,Toronto, Canada),<br>Immunoblot: SRX-101A Automatic X-ray Film Developer (Konica Minolta Medical &Graphic INC),<br>IHC staining and H&E staining images: EVOS M500 Cell Imaging System (Thermofisher),<br>DNA, RNA and protein concentrations: NanoDrop 2000 Spectrophotometer (Thermo Scientific). |
| Data analysis | Data representation and statistical analysis: Graphpad Prism 9 and Microsoft Office Excel 2016. Echocardiography measurement were analyzed using Vevo LAB software 5.6.0. Western blotting films were scanned by CanoScan 9000F Mark II. Images were processed with Adobe Photoshop CC 2019. |

For manuscripts utilizing custom algorithms or software that are central to the research but not yet described in published literature, software must be made available to editors and reviewers. We strongly encourage code deposition in a community repository (e.g. GitHub). See the Nature Portfolio guidelines for submitting code & software for further information.

## Data

Policy information about availability of data

All manuscripts must include a data availability statement. This statement should provide the following information, where applicable:

- Accession codes, unique identifiers, or web links for publicly available datasets
- A description of any restrictions on data availability
- For clinical datasets or third party data, please ensure that the statement adheres to our policy

All data used and analyzed for the associated manuscript are included in this published article (and its supplementary information files).

## Research involving human participants, their data, or biological material

Policy information about studies with human participants or human data. See also policy information about sex, gender (identity/presentation), and sexual orientation and race, ethnicity and racism.

| | |
|---|---|
| Reporting on sex and gender | N/A |
| Reporting on race, ethnicity, or other socially relevant groupings | N/A |
| Population characteristics | N/A |
| Recruitment | N/A |
| Ethics oversight | N/A |

Note that full information on the approval of the study protocol must also be provided in the manuscript.

# Field-specific reporting

Please select the one below that is the best fit for your research. If you are not sure, read the appropriate sections before making your selection.

☒ Life sciences ☐ Behavioural & social sciences ☐ Ecological, evolutionary & environmental sciences

For a reference copy of the document with all sections, see nature.com/documents/nr-reporting-summary-flat.pdf

# Life sciences study design

All studies must disclose on these points even when the disclosure is negative.

| | |
|---|---|
| Sample size | Sample sizes were chosen from past knowledge or internal pilot experiment enabling enough power to be adequate for statistical analysis. Sample sizes are indicated in figure legends or related "Methods" section. |
| Data exclusions | No data were excluded from the analyses. |
| Replication | Experiment where reproduced at least two times independently and successfully reproduced. Reproducibility of the experiments and significances of the results are described in details in Figure Legends and in the "Methods" section. |
| Randomization | No randomization was done as it is not relevant for allocation of mice. Mice were selected according to their genotype, age and sex matched. |
| Blinding | A formal blind group assignment was not applied since all data were collected independently of biased intervention. No pre-selection screening was performed other than to ensure that all experimental mice were healthy. Mice were chosen randomly for experiments during in vitro experiments and in vivo experiments. |

# Reporting for specific materials, systems and methods

We require information from authors about some types of materials, experimental systems and methods used in many studies. Here, indicate whether each material, system or method listed is relevant to your study. If you are not sure if a list item applies to your research, read the appropriate section before selecting a response.

## Materials & experimental systems

| n/a | Involved in the study | |
|---|---|---|
| | x | Antibodies |
| | x | Eukaryotic cell lines |
| x | | Palaeontology and archaeology |
| | x | Animals and other organisms |
| x | | Clinical data |
| x | | Dual use research of concern |
| x | | Plants |

## Methods

| n/a | Involved in the study | |
|---|---|---|
| x | | ChIP-seq |
| | x | Flow cytometry |
| x | | MRI-based neuroimaging |

# Antibodies

Antibodies used

Antibodies used for Western Blot (link to supplier page with detailed validation protocol):
anti-TRIM29 (1:1000; A301-210A; Bethyl)
https://www.citeab.com/antibodies/654965-a301-210-anti-trim29-atdc-polyclonal
anti-TRIM29 (1:1000; sc-33151; Santa Cruz)
https://datasheets.scbt.com/sc-33151.pdf
anti-ANP (1:1000; sc-515701; Santa Cruz)
https://www.scbt.com/p/anp-antibody-f-2
anti-FITC (1:2000; 71-1900; Thermo Fisher Scientific)
https://www.thermofisher.com/antibody/product/FITC-Antibody-Polyclonal/71-1900
antibody to phosphorylated PERK (1:1000; MA5-15033; Thermo Fisher Scientific)
https://www.thermofisher.com/antibody/product/Phospho-PERK-Thr980-Antibody-clone-G-305-4-Monoclonal/MA5-15033
anti-PERK (1:1000; 3192S; Cell Signaling Technology)
https://www.cellsignal.com/products/primary-antibodies/perk-c33e10-rabbit-mab/3192
anti-IRE1a(1:1000; 3294S; Cell Signaling Technology)
https://www.cellsignal.com/products/primary-antibodies/ire1a-14c10-rabbit-mab/3294
anti-ATF6 (1:1000; 65880S; Cell Signaling Technology)
https://www.cellsignal.com/products/primary-antibodies/atf-6-d4z8v-rabbit-mab/65880
anti-CHOP (1:1000; 2895S; Cell Signaling Technology)
https://www.cellsignal.com/products/primary-antibodies/chop-l63f7-mouse-mab/2895
anti-cleaved caspase-3 (1:1000; 9664S; Cell Signaling Technology)
https://www.cellsignal.com/products/primary-antibodies/cleaved-caspase-3-asp175-5a1e-rabbit-mab/9664
anti-BCL2 (1:1000; 3498S; Cell Signaling Technology)
https://www.cellsignal.com/products/primary-antibodies/bcl-2-d17c4-rabbit-mab/3498
anti-BAX (1:1000; 2772S; Cell Signaling Technology)
https://www.cellsignal.com/products/primary-antibodies/bax-antibody/2772
anti-STING (1:1000; 13647S; Cell Signaling Technology)
https://www.cellsignal.com/products/primary-antibodies/sting-d2p2f-rabbit-mab/13647
anti-IRF3 (1:1000; 4302S; Cell Signaling Technology)
https://www.cellsignal.com/products/primary-antibodies/irf-3-d83b9-rabbit-mab/4302
anti-TBK1 (1:1000; 3504S; Cell Signaling Technology)
https://www.cellsignal.com/products/primary-antibodies/tbk1-nak-d1b4-rabbit-mab/3504
anti-SUMO1 (1:1000; 4930S; Cell Signaling Technology)
https://www.cellsignal.com/products/primary-antibodies/sumo-1-antibody/4930
anti-SUMO2/3 (1:1000; 4971S; Cell Signaling Technology)
https://www.cellsignal.com/products/primary-antibodies/sumo-2-3-18h8-rabbit-mab/4971
antibody to phosphorylated IRF3 at Ser396 (1:1000; 4947S; Cell Signaling Technology)
https://www.cellsignal.com/products/primary-antibodies/phospho-irf-3-ser396-4d4g-rabbit-mab/4947
antibody to phosphorylated TBK1 at Ser172 (1:1000; 5483S; Cell Signaling Technology)
https://www.cellsignal.com/products/primary-antibodies/phospho-tbk1-nak-ser172-d52c2-xp-rabbit-mab/5483
antibody to phosphorylated IRE1a (1:1000; ab48187, Abcam)
https://www.abcam.com/products/primary-antibodies/ire1-phospho-s724-antibody-ab48187.html
anti-His (1:5000; 652504; BioLegend)
https://www.biolegend.com/en-us/products/hrp-anti-his-tag-antibody-9873?GroupID=BLG13798
anti-Flag (1:5000; A8592; Sigma)
https://www.sigmaaldrich.com/US/en/product/sigma/a8592
anti-GAPDH (1:20,000; G9295; Sigma)
https://www.sigmaaldrich.com/US/en/product/sigma/g9295
anti-HA (1:5000; H6533; Sigma)
https://www.sigmaaldrich.com/US/en/product/sigma/h6533
anti-Myc (1:5000; 16-213; Sigma)
https://www.emdmillipore.com/US/en/product/Anti-Myc-Tag-Antibody-clone-4A6-HRP-conjugate,MM_NF-16-213
Horseradish Peroxidase conjugated goat anti-mouse IgG, light chain specific (1:5000, 115-035-174, Jackson ImmunoResearch)

https://www.jacksonimmuno.com/catalog/products/115-035-174
Horseradish Peroxidase conjugated mouse anti-rabbit IgG, light chain specific (1:5000, 211-032-171, Jackson ImmunoResearch)
https://www.jacksonimmuno.com/catalog/products/211-032-171

Antibodies used for Immunohistochemistry (IHC):
anti-KDEL ER marker antibody (1:400; sc-58774, Santa Cruz)
https://www.scbt.com/p/kdel-er-marker-antibody-10c3
anti-TRIM29 (1:400; sc-33151; Santa Cruz)
https://datasheets.scbt.com/sc-33151.pdf

Antibodies used for cytometry
APC/Cyanine7 anti-mouse CD45 antibody (103116, BioLegend)
https://www.biolegend.com/en-us/cellular-dyes-and-ancillary-products/apc-cyanine7-anti-mouse-cd45-antibody-2530
Brilliant Violet 785 anti-mouse/human CD11b antibody (101243, BioLegend)
https://www.biolegend.com/en-us/products/brilliant-violet-785-anti-mouse-human-cd11b-antibody-7958
APC anti-mouse Gr1 antibody (108412, BioLegend)
https://www.biolegend.com/en-us/products/apc-anti-mouse-ly-6g-ly-6c-gr-1-antibody-456
PE anti-mouse Ly6C antibody (128008, BioLegend)
https://www.biolegend.com/en-us/products/pe-anti-mouse-ly-6c-antibody-4904
FITC anti-mouse Ly6G antibody (127606, BioLegend)
https://www.biolegend.com/en-us/products/fitc-anti-mouse-ly-6g-antibody-4775
Brilliant Violet 421 anti-mouse CD64 antibody (164407, BioLegend)
https://www.biolegend.com/en-us/products/brilliant-violet-421-anti-mouse-cd64-fcgammari-antibody-23594
FITC anti-mouse CD3 antibody (100204, BioLegend)
https://www.biolegend.com/en-us/products/fitc-anti-mouse-cd3-antibody-45
PE anti-mouse CD19 antibody (152408, BioLegend)
https://www.biolegend.com/en-us/products/pe-anti-mouse-cd19-antibody-13641
PE/Cyanine7 anti-mouse CD4 antibody (100528, Biolegend)
https://www.biolegend.com/en-us/products/pe-cyanine7-anti-mouse-cd4-antibody-1932
PerCP/Cyanine5.5 anti-mouse CD8a antibody (100734, BioLegend)
https://www.biolegend.com/en-us/products/percp-cyanine5-5-anti-mouse-cd8a-antibody-4255
APC anti-mouse IFN-γ antibody (505810, BioLegend)
https://www.biolegend.com/en-us/products/apc-anti-mouse-ifn-gamma-antibody-993

Validation

All antibodies used in this study are commercially available and have been validated and commonly used in the field. The manufacturer' websites provide details regarding validations and associated reference publication (see above section). Additionally, all antibodies have been validated by lab personnel with proper isotype control (for FACS staining) or by identification of specific signal at appropriate size (for Immunoblot experiment).

## Eukaryotic cell lines

Policy information about cell lines and Sex and Gender in Research

Cell line source(s)

HEK 293T, HEK 293FT and HeLa cell lines were purchased from ATCC. The AC16 human cardiomyocytes were purchased from EMD Millipore.

Authentication

All cell lines were authenticated on their morphology and growth.

Mycoplasma contamination

All cell lines were tested and free of mycoplasma contamination.

Commonly misidentified lines
(See ICLAC register)

No commonly misidentified cell lines were used.

## Animals and other research organisms

Policy information about studies involving animals; ARRIVE guidelines recommended for reporting animal research, and Sex and Gender in Research

Laboratory animals

Trim29fl/fl C57BL/6J mice were generated as described (Dou et al. 2019). C57BL/6J αMyHC-Cre transgenic mice was obtained from Jackson Laboratory. Both male and female mice between 6-8 weeks old were used. Animals were housed under the following conditions: temperatures of 68-72F, 30-70% humidity, 10-15 fresh air exchanges hourly, and a 12:12h light:dark cycle (lights were on from 07:00-19:00). Mice were housed in sterile individually-ventilated cages (Techniplast S.p.A., Buguggiate, Italy) containing autoclaved Bed-o'Cobs 1/4" bedding (The Andersons, Inc.), a sterile cotton nesting square or sterile crinkle nesting material, and received approximately 75 air changes hourly. Mice were housed at a density of up to five mice.

Wild animals

The study did not involve wild animals.

Reporting on sex

In the study design, male mice were selected primarily based on the following considerations: 1) mortality and morbidity of cardiovascular diseases are sex-dependent (Circulation. 2019 Feb 19,139(8):1025-1035, PMID: 30779652). 2) Studies consistently

report that myocarditis occurs more often in men than women (Front Cardiovasc Med. 2023 Mar 2,10:1129348, PMID: 36937911; Curr Opin Physiol. 2023 Oct, 35:10070, PMID: 37662585). 3) Studies, including our previous publication, consistently report that male mice develop worse myocarditis than female mice in CVB3 induced viral myocarditis mouse model (Infect Immun. 1981 Apr, 32 (1):68-7, PMID: 6260687; Front Cardiovasc Med. 2023 Mar 2,10:1129348, PMID: 36937911; J Biomed Sci. 2022 Jul 31,29(1):55, PMID: 35909127). Therefore, only male mice were used in our animal studies. Both sexes of neonatal mice were used to isolate neonatal cardiomyocytes since it is difficult to separate male and female neonates.

| | |
|---|---|
| Field-collected samples | No field collected samples were used. |
| Ethics oversight | All animal studies were ethically reviewed and approved by the Houston Methodist Animal Care Committee (IS00005756) and were carried out in accordance with the National Institutes of Health Guidelines for the Care and Use of Laboratory Animals. |

Note that full information on the approval of the study protocol must also be provided in the manuscript.

## Plants

| | |
|---|---|
| Seed stocks | N/A |
| Novel plant genotypes | N/A |
| Authentication | N/A |

## Flow Cytometry

### Plots

Confirm that:

- [x] The axis labels state the marker and fluorochrome used (e.g. CD4-FITC).
- [x] The axis scales are clearly visible. Include numbers along axes only for bottom left plot of group (a 'group' is an analysis of identical markers).
- [x] All plots are contour plots with outliers or pseudocolor plots.
- [x] A numerical value for number of cells or percentage (with statistics) is provided.

### Methodology

| | |
|---|---|
| Sample preparation | Preparation of cells from spleen was done by mechanical disaggregation of the tissue through a 100um strainer using a syringe plunger. Cell suspension was then moved to a collection tube and the 70 µm cell strainer was washed twice with ice-cold PBS, followed by incubation in red blood cell lysis buffer (RBCLB) to remove the erythrocytes from the cell suspension. For each spleen, 1ml of room temperature RBCLB was added and the tube shaken manually for 2min before washing with ice-cold PBS. The cell suspensions was then used for flow cytometry.<br>Hearts were perfused with PBS and then minced using a razor blade. The minced hearts were then incubated with 2 mL of tissue digestion enzyme solution with 3,000 U/ml Collagenase II and 90 U/ml DNase I (Sigma) for 30 minutes at 37°C in 35mm dishes. After incubation with digestion enzymes, tissues were dissociated using gentleMACS Dissociator (Miltenyi). Cells were washed and filtered through 70 µm cell strainers. The filtered cells were suspended in 3ml 40% Percoll and centrifuged at 2200rpm (Acceleration:6,Deceleration:1) for 10 minutes at room temperature for isolating heart infiltrated mononuclear cells. The cell suspensions was then used for flow cytometry. |
| Instrument | LSR-II flow cytometer (Beckton Dickinson) |
| Software | FACS data were analyzed using FlowJo v10 software (Tree Start). |
| Cell population abundance | No sorted samples were used. |
| Gating strategy | We have provided the gating strategy in Supplementary figure, providing all the necessary details.<br>FSC-A/SSC-A was used to identify the lymphocytes by size. FSC-A/FSC-H followed by FSC-A/SSC-A were used to define singlets. Zombie Aqua fixable viability was used to identify live cells. APC/Cyanine7 anti-mouse CD45 antibody was used to identify CD45 positive cells. MDSC were identified as CD11b+Gr1+ cells. The mMDSC were identified as CD11b+, Gr1+, Ly6C+ and Ly6G- cells. Macrophages were identified as CD11b+CD64+ cells. Neutrophils were identified as CD11b+Ly6G+ cells. B cells were identified as CD3-CD19+ cells. T cells were identified as CD3+ cells. CD4 and CD8 T cells were selected by the expression of CD4 and CD8, respectively. IFN-r producing CD8+ T cells were identified as IFN-r+ CD8+ T cells. |

- [x] Tick this box to confirm that a figure exemplifying the gating strategy is provided in the Supplementary Information.
